# Testosterone Therapy, Thrombophilia, Venous Thromboembolism, and Thrombotic Events

**DOI:** 10.3390/jcm8010011

**Published:** 2018-12-21

**Authors:** Charles J. Glueck, Naila Goldenberg, Ping Wang

**Affiliations:** Cholesterol, Metabolism, and Thrombosis Center, Cincinnati Ohio, OH 45220, USA; Aliandlog@yahoo.com (N.G.); Dz2335@yahoo.com (P.W.)

**Keywords:** testosterone, thrombophilia, hypofibrinolysis, venous thromboembolism (VTE), Factor V Leiden heterozygosity, lupus anticoagulant, lipoprotein (a), thrombosis

## Abstract

In our sequential studies of 67 and 21 patients, testosterone therapy (TT) interacted with thrombophilia–hypofibrinolysis, leading to venous thromboembolism (VTE). Compared to 111 VTE controls not taking TT (VTE-no TT), the 67 and 21 cases were more likely (*p* < 0.05 for all) to have Factor V Leiden (FVL) heterogeneity (24% and 33% vs. 12%), the lupus anticoagulant (14% and 33% vs. 4%), and high lipoprotein(a) (33% vs. 13%, *n* = 21). After a first VTE and continuing TT, 11 thrombophilic cases had a second VTE despite adequate anticoagulation, 6 of whom, still anticoagulated, had a third VTE. The greatest density of thrombotic events was at three months after starting TT, with a rapid decline by 10 months. From <1 to 8 months after starting TT, 65% of VTE occurred, which may reflect TT-induced depletion of susceptible thrombophilic patients, leaving a winnowed residual group with fewer VTE events despite the continuation of TT. Before starting TT, we suggest screening for FVL, lipoprotein(a), and the lupus anticoagulant to identify patients at increased VTE risk, with an adverse risk-to-benefit ratio for TT. We suggest that TT should not be started in patients with known thrombophilia–hypofibrinolysis, and should not be continued after a first VTE. When TT is given to patients with thrombophilia–hypofibrinolysis, VTE may occur and then recur despite adequate anticoagulation.

## 1. Introduction

In June 2014, based on post-marketing surveillance reports including citation of some of our studies [1], the U.S. Food and Drug Administration FDA [2] and Canada Health [3] added a warning regarding the risks of venous thromboembolism (VTE) to the label of all testosterone products. In January 2016, the FDA released a warning of possible increased cardiovascular risk (heart attack and stroke) associated with testosterone therapy (TT) [4]. The FDA has subsequently emphasized the importance of a prospective, blinded, placebo-controlled clinical trial to assess cardiovascular and thrombotic safety of TT [5].

VTE, particularly pulmonary embolism (PE), is associated with significant mortality risk [6], is a major component of hospital expense [7], and is associated with a 52% increased risk of subsequent work disability [8].

The sole currently available placebo-controlled clinical trial is the National Institutes of Health-supported prospective placebo-controlled Testosterone Trial of 790 men over age 65 with low serum testosterone levels (<275 ng/dL) and evidence of sexual dysfunction, physical dysfunction, or reduced vitality [9]. Men in the intervention arm received 12 months of testosterone gel therapy to increase T levels to the mid-normal range for men aged 19–40. Compared to the placebo, the TT group reported small increases in sexual activity, desire, and erectile function, and slightly improved mood, but no differences in 6-min walking distance or vitality. In four additional publications from the placebo-controlled trial, TT had no effect on memory impairment, but increased noncalcified coronary plaque volume on computed tomography CT angiography and increased bone mineral density and hemoglobin levels. The follow-up period was not long enough or powered to measure differences in cardiovascular events, fractures, or all-cause mortality [10,11,12,13].

Ultimately, the risk-to-benefit ratio of TT could optimally be explained by a placebo-controlled clinical trial comparable to the Women’s Health Initiative trials [14], which were required to resolve conflicting epidemiological and clinical data.

## 2. Materials and Methods

Our studies of 67 and 21 patients with VTE after starting TT, and in 111 controls with VTE not taking TT (VTE-no TT), were carried out with signed informed consent, following protocols approved by our institutional Institutional Review Board IRB [15,16], as were our other studies of TT, thrombophilia, and thrombosis [17,18].

### 2.1. Study Entry Criteria

We excluded from entry into the studies both TT patients and VTE-no TT controls whose VTE was provoked, associated with cancer, polycythemia vera, recent soft tissue trauma, bone fracture, hip-knee-foot surgery, airline flights >8 hours, or immobilization for >2 weeks. Hence, by selection, thrombotic events, both in TT cases and in VTE-no TT controls entered into the study, occurred spontaneously without superimposed acute triggering factors.

### 2.2. Study Design, Cases, and Controls

The 88 patients and 111 VTE-no TT controls were studied in their serial order of referral over the past 4 years to our thrombosis center by their family physicians for evaluation of thrombophilia–hypofibrinolysis as a pathoetiology of their venous thrombotic events. TT had been prescribed by the referring physicians, and if not already stopped, it was discontinued by us before our assessment of thrombophilia–hypofibrinolysis. Measurements of thrombophilia–hypofibrinolysis were carried out at least 2 months after the cases’ initial thrombotic event.

Because we did not initiate TT in the 88 TT-treated patients with VTE, we did not know the etiology of hypogonadism in the TT-treated group.

Our studies of 88 patients with thrombotic events after starting TT along with 111 VTE-no TT controls represent a clinical case-control series examining interactions between TT and underlying familial and acquired thrombophilia–hypofibrinolysis in patients referred by virtue of unprovoked venous thrombotic events. These studies amplify findings from our earlier publication [1], which was cited by the U.S. FDA and Canada Health in mandating a warning regarding the risks of VTE on the label of all testosterone products [4,19]. On a population basis, rather than our case-control series, population thrombophilia studies would need to be done in a cohort of men receiving TT with and without VTE and in men without TT exposure. Optimally, to assess the risk of VTE events in testosterone-treated hypogonadal men, and interactions of TT with underlying familial and acquired thrombophilia with VTE, a placebo-controlled double-blind trial [5] similar to the Women’s Health Trial [14,20], with assessment of thrombophilia at entry, will be required.

### 2.3. Statistical Analysis

TT case versus VTE-no TT control comparisons of coagulation factors were made both by Mantel–Haenszel χ^2^ tests and by stepwise logistic regression after adjusting for age and gender.

### 2.4. Laboratory Measures of Thrombophilia and Hypofibrinolysis

In the 88 patients and 111 VTE-no TT controls, laboratory assessment of thrombophilia–hypofibrinolysis included the following assays.

#### 2.4.1. PCR Assays

PCR measures of thrombophilia (G1691A Factor V Leiden, G20210A prothrombin, methylenetetrahyrofolate reductase (MTHFR) C677T/A198C) and hypofibrinolysis (plasminogen activator inhibitor (PAI-1 SERPINE 1) gene polymorphism 4G/5G) were performed in all cases and controls using previously published methods [21,22] by laboratory staff blinded to the participants’ status (diagnosis, severity of disease).

#### 2.4.2. Serologic Measures of Thrombophilia and Hypofibrinolysis

Serologic measures of thrombophilia included Factors VIII and XI, homocysteine, antigenic proteins antithrombin III, C, S, and free S, as well as the antiphospholipid antibody APL syndrome (anticardiolipin antibodies (ACLA IgG, IgM), the lupus anticoagulant, anti-beta2-glycoprotein). Established, previously published methods were used [22]. High homocysteine was identified by levels > the laboratory 95th percentile [23]. The three components of the antiphospholipid antibody syndrome were measured by ELISA using previously published methods [24,25,26].

Plasminogen activator inhibitor-1 activity levels, associated with hypofibrinolysis, were not measured.

## 3. Results

### 3.1. Sixty-Seven Patients with Thrombotic Events Six Months (Median) after Starting Testosterone Therapy

In 2016 [15], we assessed thrombotic events after starting TT in 67 patients, 59 men and 8 women, comparing them to 111 patient controls having unprovoked VTE without TT (VTE-no TT controls). None of the 67 patients had sustained thrombotic events before starting TT. In the 67 patients, 47 had deep venous thrombosis (DVT-PE) [19,21,27], 16 had osteonecrosis, and 4 had ocular thrombosis.

Mean ± SD age was 57 ± 14 years. Testosterone gel (50 mg/day) was used by 58%, intramuscular T (150–200 mg/week) by 31%, human chorionic gonadotropin HCG by 2%, a patch by 3%, pellets by 4%, and clomiphene by 1%. Of the 67 patients, 70% had VTE (without arterial thrombosis), 24% had osteonecrosis, and 6% had ocular (retinal vein) thrombosis. Mean ± SD duration of testosterone therapy before the occurrence of a thrombotic event was a median of 6 months, a mean ± SD of 10.7 ± 13.3 months, and a 25th–75th percentile of 3–12 months [15]. The time course between starting TT and the development of a thrombotic event (6 months) was comparable to VTE in women on hormone replacement therapy, where VTE rates are highest in the first year of treatment and are much more pronounced in women at high risk for VTE because of thrombophilias [28].

Cases differed from controls for Factor V Leiden (FVL) heterozygosity (16/67 cases (24%) vs. 13/111 VTE-no TT controls (12%), *p* = 0.038) and for the lupus anticoagulant (9/64 cases (14%) vs. 4/106 (4%) VTE-no TT controls, *p* = 0.019). Our findings that 24% of our TT-VTE patients had FVL heterogeneity versus 12% of our VTE-no TT controls emphasized the importance of FVL mutations for VTE [29,30] and the interaction of FVL with TT, promoting thrombotic events.

### 3.2. Twenty-One Men with Thrombotic Events Six Months (Median) after Starting Testosterone Therapy

In 2018 [16], we reported thrombotic events after starting TT in 21 men who sustained 23 VTEs. These 21 patients were referred to us and were studied sequentially after our first 67 patients [15]. None of the 21 patients had sustained thrombotic events before starting TT. Of the 21 patients, 8 had DVT alone, 5 had DVT and PE, 3 had PE alone, 2 had ischemic stroke, 2 had idiopathic osteonecrosis, and 1 had central retinal artery thrombosis.

Mean ± SD age was 52 ± 14 years. Ten men had been given TT intramuscularly (~200 mg/week), 7 used TT gel (50 mg/day), 3 clomiphene, and 1 HCG. The median duration of therapy was 6 months, and 76% of the men received TT between <1 and <8 months before sustaining a thrombotic event.

The density of thrombotic events was greatest at 3 months after starting TT, with a rapid decline in events by 10 months. Finding a peak of VTE events at 3 months after starting TT was entirely congruent with the population study by Martinez et al. [31].

The 21 cases with VTE on TT [16] differed from 110 patient controls with unprovoked VTE, not taking TT (VTE-no TT controls) for FVL heterozygosity (33% vs. 13%, *p* = 0.037), for high lipoprotein(a) (Lp(a)) (55% vs. 17%, *p* = 0.012), and for the lupus anticoagulant (33% vs. 4%, *p* = 0.003). These differences between cases and VTE-no TT controls were independent of age and gender. These results were congruent with our 2016 report on 67 patients [15], where 24% of cases had FVL heterozygosity and 14% had the lupus anticoagulant. As in 2016 [15], we concluded that TT can interact with underlying thrombophilia–hypofibrinolysis, promoting VTE. We suggested that TT should not be started in subjects with known thrombophilia. We recommended that coagulation screening, particularly for FVL, lipoprotein(a), and the lupus anticoagulant should be considered before starting TT, to identify men at high VTE risk who have an adverse risk-to-benefit ratio for TT.

### 3.3. Pooled Analysis of 88 Cases

The results from our studies of 21 [16] and 67 cases [15] with VTEs on TT were pooled (*n* = 88), since they were sequential but separate sets of patients without overlap. These two studies had the same inclusion and exclusion criteria, did not differ by age or gender (*p* > 0.05), did not differ by familial or acquired thrombophilia characteristics (*p* > 0.05), followed the same protocol in in the same thrombosis research center, and used the same coagulation laboratory, the same control group, and the same investigator group. None of the 88 patients had sustained thrombotic events before starting TT.

Mean ± SD age in the 79 men was 52.4 ± 14.1, and in the 9 women, 51.8 ± 8.1 years. In the 79 men (of 88 cases), 41 used T gel (50 mg/day), 30 intramuscular T (150–200 mg/week), 4 clomiphene, 3 HCG, and 1 patch. In the 9 women, 3 used T gel (50 mg/day), 3 subcutaneous T pellet (75 mg T, 75 mg estradiol (E2)), 2 intramuscular (100 mg/week), and 1 patch (300 ug/day).

In 29 of the 79 men in our two studies [15,16], we were able to recover measures of serum total and free testosterone and estradiol (E2), which had been obtained before TT and subsequently after a median of 6 months on TT (Table 1). Two-thirds of the men had low T and free T before TT and one-third had normal levels (Table 1). On TT, the percentage of men with low serum T and free T fell to 28% and 36%, respectively, and 62% of men achieved normal T on TT (Table 1). Before TT, 13% of men had high E2, and this rose to 24% on TT (Table 1). We have speculated [1] that high E2 levels during testosterone therapy in men may contribute to thrombotic events via interactions with familial and acquired thrombophilias as exogenous testosterone is aromatized to E2.

The case-control comparisons for FVL heterozygosity in the 67 and 21 case groups did not differ (*p* > 0.05), with odds ratios (ORs) and 95% confidence intervals (CIs) of 2.37 (1.6–5.3) and 3.42 (1.1–10.5), respectively. For all 88 cases, pooled, the cases were more likely than 110 VTE-no TT controls to have FVL heterozygosity (22 of 85 (27%) vs. 14/110 (13%), Mantel–Haenszel χ^2^ OR 2.51 (95% CI 1.28–4.44), *p* = 0.004). The case-control comparisons for the lupus anticoagulant in the 67 and 21 case groups did not differ ((*p* > 0.05), OR 4.17 (1.23–14.2) and 10.8 (2.53–47.2)). In the pooled 88 cases, cases were more likely to have the lupus anticoagulant (14 of 79 TT cases (18%) vs. VTE-no TT controls 4 of 91 (4%)). For all 88 cases pooled, the case-control Mantel–Haenszel odds ratio for the lupus anticoagulant in cases versus controls was OR 5.09, 95% CI (1.89–13.98), *p* < 0.0001.

By stepwise logistic regression after covariance adjusting for age and gender, cases were more likely to have Factor V Leiden heterogeneity than VTE-no TT controls (OR 2.3 (95% CI 0.99–5.36), *p* = 0.053). By stepwise logistic regression after adjusting for age and gender, cases were more likely than controls to have either Factor V Leiden heterogeneity or the lupus anticoagulant than VTE-no TT controls (OR 3.13 (95% CI 1.46–6.68), *p* = 0.003). By stepwise logistic regression, neither Lp(a) (*p* = 0.098) or the lupus anticoagulant (*p* = 0.13) differed between cases versus controls. In all models, age and gender were not significant (*p* > 0.10).

**Figure 1:**
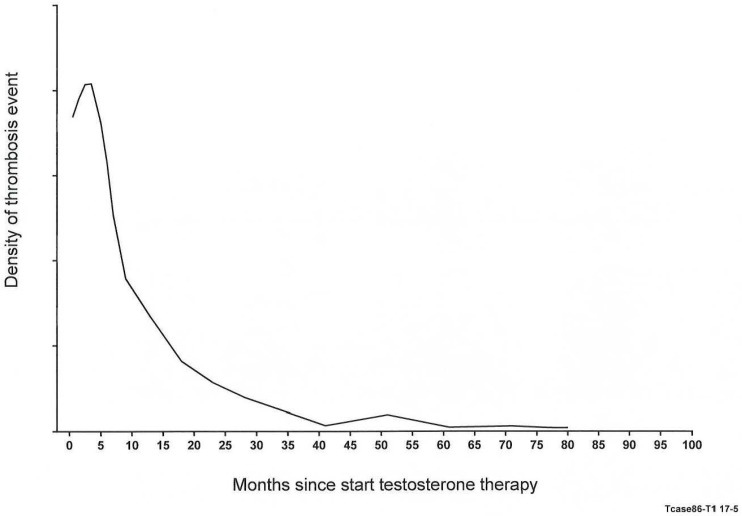
Quadratic spline curve illustrating the density of venous thromboembolism (VTE) events over time since starting testosterone therapy (*n* = 88).

An important new finding in our study of 88 patients, paralleling Martinez et al. [31], was that VTE events peaked at 3 months, with a subsequent rapid decline in the density of events by 10 months (Figure 1). For all 88 TT-VTE cases, the density of the timing of VTE was graphed, and a quadratic spline curve was fitted (Figure 1). As displayed in Figure 1, the greatest density of thrombotic events was observed at 3 months after starting TT, with a rapid decline in density of events by 10 months. Sixty-five percent of the thrombotic events occurred in a time frame ranging from <1 month to 8 months after starting TT.

**Figure 2:**
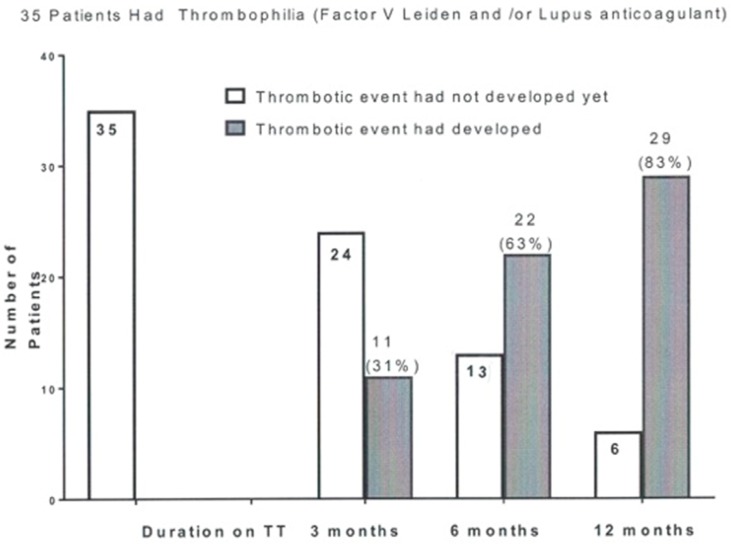
Depletion from the group of 35 thrombophilic patients (Factor V Leiden heterozygosity and/or the lupus anticoagulant) susceptible to developing a thrombotic event 3, 6, and 12 months after starting testosterone therapy.

As displayed in Figure 2, of the 88 patients, there were 35 who had FVL heterozygosity and/or the lupus anticoagulant. After 3, 6, and 12 months on TT, 11 (31%), 22 (63%), and 29 patients (83%) were removed from the cohort, having sustained VTE events (Figure 2). This removal of susceptible subjects [32] may account for the peaking of VTE event rates at 3 to 6 months, followed by a rapid decline (Figure 1). Sixty-five percent of the thrombotic events occurred in a time frame ranging from <1 month to 8 months after starting TT. Martinez et al. [31] have also commented that the pattern of VTE events peaking rapidly in the first 3 months and declining gradually thereafter [31] may account for the underestimation of the association between TT use and VTE, as in a recent case-control study [33]. This would leave over time a thrombophilia-winnowed residual group with progressively fewer VTE events (Figure 2).

In the current report, for the pooled 88 patients, there were similar time intervals between starting TT and VTE events peaking at 3 months and cardiovascular disease (CVD) events peaking ~3 months after starting TT [34]. We speculate that the short 3-month interval between starting TT and the development of both thrombotic and CVD events [34,35,36,37] may indicate a shared thrombotic pathophysiology, since CVD events occurring ~3 months after starting TT cannot reflect a conventional arterial atherosclerotic event. Within this frame of reference, over an average follow-up of 7.4 years in a retrospective study of androgenic anabolic steroid use, mortality was three times higher in Danish men who used androgenic anabolic steroids than among nonuser controls, with a hazard ratio of 3.0 and a 95% CI of 1.3–7 [38].

### 3.4. Recurrent Thrombotic Events when Testosterone Therapy Is Continued after a First Event, Despite Adequate Anticoagulation

After a first thrombotic event and continuing TT [15], 11 of 67 patients, all thrombophilic, had a second thrombotic event despite adequate anticoagulation with warfarin (*n* = 9) or novel oral anticoagulants (NOWACs) (*n* = 2), 6 of whom, still anticoagulated (4 on warfarin, 2 on NOWACs), had a third thrombosis. Similarly, Colburn et al. have described recurrent renal infarctions in a patient taking both testosterone and anabolic steroids despite anticoagulation with apixaban [39]. After a thrombotic event, if TT is continued in thrombophilic patients, concomitant and adequate anticoagulation does not appear to prevent recurrent thrombotic events [15,40,41], and TT should be stopped.

### 3.5. Thrombotic Events in Nine Women at a Median of Three Months after Starting Testosterone Therapy

In our 67-patient study [15], there were 8 women, and we recently added 1 previously unpublished case. All 9 women were given TT to improve libido, and in 1 of the 9, also for anabolic effect. None of the 9 women had sustained thrombotic events before starting TT. Thrombotic events occurred at a median of 3 months after starting TT. In the 9 women, 3 used T gel (50 mg/day), 3 subcutaneous T pellets (75 mg T, 75 mg E2), 2 intramuscular (100 mg/week), and 1 used a patch (300 ug/day).

On TT, all 9 women had high serum T (>UNL 48 ng/dL, mean 160 ± 101, median 116, range 76–402 ng/dL). Two women were FVL heterozygotes, 2 prothrombin gene 202010A heterozygotes, 1 had high Factors VIII (346%, UNL 150%) and XI (154%, UNL150%), 2 had high anticardiolipin antibody ACLA IgM, 1 had high homocysteine, 1 had high Lp(a), and 1 had low antigenic protein C.

By logistic regression, after adjusting for age, the 9 women differed from 110 normal controls for FVL heterozygosity (OR 17.7, 95% CI 2.02–155.3), for prothrombin gene heterozygosity (OR 11.72, 95% CI 1.51–90.9), and for high anticardiolipin antibody IgM (OR 16.7, 95% CI 1.9–146.2).

Considering that many [42,43,44], but not all [45,46,47], blinded controlled clinical trials have documented limited to no efficacy for TT in significantly enhancing female libido, the risk-to-benefit ratio of TT in women is high [48]. The risk for developing thrombotic events is particularly higher in women with underlying familial and acquired thrombophilia who undergo TT [15]. Since TT in this and other studies [48] has increased the risk of VTE, women should be warned of VTE risk before contemplating TT, given a very adverse risk-to-benefit ratio.

### 3.6. Osteonecrosis Developing at a Median of Six Months after Starting Testosterone Therapy in 16 Patients

Among the 67 patients with thrombotic events after starting TT [15], 16 (12 men, 4 women) developed idiopathic osteonecrosis 6 months (median) after the onset of TT [18]. None of the 16 patients had osteonecrosis before starting TT. Idiopathic osteonecrosis, with intra-osseous venous thrombosis, is often caused by thrombophilia [21,27,49], often develops after starting TT, and its progression may be slowed or stopped by discontinuation of TT, and, thereafter, anticoagulation [18,50,51,52,53]. Of the 16 cases, 5 (31%) were FVL heterozygotes versus 4 of 48 patients who had osteonecrosis and were not receiving TT (*p* = 0.04). Subsequently, we reported 2 additional cases [50] of osteonecrosis after starting TT, both found to be heterozygous for the FVL mutation.

### 3.7. Thrombotic Events in Six Patients with Klinefelter Syndrome

Of the 80 men we have studied, we have previously described DVT, PE, and mesenteric artery thrombosis in 6 with Klinefelter syndrome [17], 4 of whom had high Factor VIII, 3 of whom had high Factor XI, and 1 who was heterozygous for the G20210A Prothrombin gene mutation. We have speculated that the previously known increased rate of DVT-PE and other thrombotic events in Klinefelter syndrome reflected an interaction between pro-thrombotic, long-term TT with previously undiagnosed familial thrombophilia [17]. Zoller has concluded that Klinefelter syndrome should be considered a genetic hypercoagulable state and is an important risk factor for VTE [54].

## 4. Discussion

In our 67- [15] and 21-patient studies [16], we concluded that screening for thrombophilia before starting TT should identify men and women at high risk for thrombotic events with an adverse risk-to-benefit ratio for TT. We also suggested that after an initial thrombotic event on TT in patients shown to have thrombophilia, TT should be stopped, given second and third thrombotic events (despite the continuation of adequate anticoagulation) if TT was continued. Similarly, Corona et al. [55] have recommended an anamnestic screening for thrombophilia before starting TT, paralleling screening before starting estrogen-progestin oral contraceptives. Moreover, pretreatment thrombophilia screening has been advocated before giving estrogen to women [28,56] [57,58]. We have concluded that thrombophilia screening before starting TT, and after an initial thrombotic event on TT, should allow a much more accurate stratification of risk [59] and allow patients to participate in informed decision making where basic preventive measures can be taken to reduce the risk of morbidity and mortality [31].

There are major long-term health consequences and economic ramifications for VTE. Aggregate costs associated with VTE treatment have been estimated to be between $375,000 to $421,000 per patient [60]. Many VTE patients develop recurrent VTE within five years of their first event [61] and require long-term anticoagulation, with estimated costs in the first year of treatment between $12,000–$15,000. Laboratory costs for thrombophilia screening in patients before starting TT need to be balanced by recognition of the very serious health consequences and costs of VTE. However, in the U.S., FVL and Prothrombin G20210A genotypes can be obtained directly by the patient from 23 and Me for ~$200, and from Kailos Genetics for ~$100 (https://www.kailosgenetics.com/buy-gene-test).

There have been two major observational population studies of VTE after starting TT: Martinez et al. [31] reported a significant TT-VTE relationship in men taking TT for ≤6 months or less, while Baillargeon et al. [33] found no significant relationship. Martinez et al. [31] studied 19,215 patients with confirmed VTE and 909,530 subjects without VTE from the same U.K. primary care database. The adjusted rate ratio of VTE for current versus no TT was 1.25, 95% CI 0.94–1.44. However, when the data were limited to the first 6 months of TT, the rate ratio of VTE was 1.63 (95% CI 1.12–2.37). This corresponded to 10.0 (1.9 to 21.6) additional VTE events above the base rate of 15.8/10,000 person years [31]. For subjects on TT without a formal diagnosis of pathological hypogonadism, the rate of VTE was higher than those with the diagnosis (OR 1.88 vs. 1.52). VTE risk was higher in those without known VTE risk factors (OR 1.91, 95% CI 1.13–3.23) versus those with known risk factors (OR 1.41, 95% CI 0.82–2.41). Despite more than 19,000 men studied, there were only 69 men with VTE on TT (0.36%). Martinez [31] noted that “…on an absolute population risk basis, the baseline and testosterone-induced increases in risks of venous thromboembolism of middle aged and older men are quantitatively comparable to the baseline (non-user) risks of venous thromboembolism in women and the increase among users of oral estrogens in combined oral contraceptives or estrogen replacement therapy for menopause.” The early increase in risk for VTE in the first 6 months on TT is also similar to that seen with oral estrogen [62].

In contrast to Martinez et al. [31], Baillargeon et al. [33] found no significant relationship between TT and VTE in a population study. Baillargeon et al. [33] studied 30,572 men ≥age 40 in a large commercial insurance program who had a primary diagnosis of VTE and received an anticoagulant in the 60 days after their diagnosis. Cases were matched with controls who had neither DVT nor PE at any time during the study period. After adjustment for cofounders, TT was not associated with an increased risk of VTE (adjusted ratio 0.90, 95% CI 0.73–1.12), an outcome entirely different from that of Martinez et al. [31]. Martinez et al. [31] noted that the fact that VTE risk peaked rapidly in the first three months and declined gradually thereafter indicates that failure to investigate the timing and duration of TT could have resulted in underestimation of the association between TT use and VTE venous thromboembolism by Baillargeon et al. [33] if overall risk estimates were based on a high proportion of patients carrying a small risk, a phenomenon known as depletion of susceptibles [32]. This could explain the discrepancy between the findings of Martinez et al. [31] and Baillargeon et al. [33]. In a subsequent meta-analysis of these two studies by Corona et al. [55], TT was not associated with an increased risk of VTE, either unadjusted (OR 1.3, 95% CI 0.66–2.55) or fully adjusted (OR 1.05, 95% CI 0.76–1.44).

In contrast to the positive association of TT with VTE reported by Martinez et al. [31], Li [63] carried out a retrospective analysis of 102,650 patients treated with exogenous testosterone and 102,650 untreated patients. No significant association was found between TT and idiopathic or overall VTE events. However, there were discrepant findings for injectable formulations and the risk of overall VTE. Ramasamy [64] carried out a retrospective chart review of 217 hypogonadal men >65 years, comparing those receiving TT (*n* = 153) to those without TT. There were four thrombotic events, all occurring after two or more years of follow-up, with no difference between TT-treated men and untreated hypogonadal men for MI, TIA/CVA, or PE. Sharma [65], in a retrospective cohort study of 10,854 veterans not receiving testosterone replacement therapy (TRT) and 60,553 receiving TRT, did not detect a significant association between TRT and the risk of DVT-PE in adult men with low serum T who were at low-to-moderate baseline risk of DVT-PE.

As emphasized by Corona et al. [55], there have been, as yet, no randomized, placebo-controlled trials (RCT) of TT therapy with VTE or CVD as the primary endpoint. In a meta-analysis of the limited RCT TT data, Xu et al. [66], using a fixed effect model, reported that TT was associated with a fivefold increased risk of VTE, based on three randomized controlled trials RCTs including 516 men. Recently, Corona et al. [55] carried out an updated systematic review and meta-analysis of RCTs on TT, including studies that reported VTE events in TT or placebo arms. Six studies were included in this meta-analysis, including 1217 and 1166 in the TT and placebo groups, with a mean trial duration of 42 weeks and a mean age of 47 years. By applying a random effect model, the use of TT was not associated with any difference in VTE in TT versus placebo groups (OR 1.96, 95% CI 0.75–5.17, *p* = 0.17).

In a recent systemic review and meta-analysis of TT and VTE, Houghton et al. [67] concluded that “…the current evidence is of low certainty but does not support an association between testosterone use and VTE in men”. However, when they performed an additional analysis stratified by hypogonadism, TT was significantly associated with VTE in men with and without a diagnosis or hypogonadism (OR 1.57, 95% CI 1.27–1.95 and 1.94, 95% CI 1.26–2.99).

Without large randomized trials of men, a “Men’s Health Initiative (MHI)” powered to assess the risk of cardiovascular disease, VTE, prostate cancer, and all-cause mortality of testosterone replacement therapy, there will be persistent controversy over whether TT is good, bad, or indeterminate. An optimal study, paralleling the Women’s Health Initiative WHI studies in women [14,20], would be prospective, placebo-controlled, and double-blind [5]. Such a trial would be difficult to fund and carry out, since Onasanya [68] concluded that any randomized controlled clinical trial aimed at detecting a difference in cardiovascular risk between TT and placebo groups would require at least 17,664 participants in each trial group. Houghton et al. [67] similarly calculated that 15,613 subjects would be required in TT and placebo groups, and noted that currently available randomized studies may simply be underpowered to detect a VTE–testosterone relationship. However, since, as in the current study and in the report by Martinez [31], the peak in VTE events was three months after starting TT, a placebo-controlled TT study might have to be only one year long to capture most of the VTE events, and, probably, cardiovascular events, the majority of which might occur within the first year of starting TT [35,36,37].

## 5. Conclusions

Our two recent studies of 88 patients (67 [15] and 21 [16] altogether) revealed VTE associated with FVL heterozygosity, the lupus anticoagulant, and lipoprotein(a), peaking three months after starting TT. These studies provide further evidence altogether congruent with the 2014 FDA warning on the TT-associated risks of VTE [2]. We suggest that TT should not be started in patients with known familial or acquired thrombophilia, and recommend screening for familial and acquired thrombophilia, particularly FVL and the lupus anticoagulant, before starting TT, to identify men and women at high risk for VTE with an adverse risk-to-benefit ratio for TT. When TT is given to patients with familial and acquired thrombophilia, thrombosis may occur and recur despite adequate anticoagulation if TT is continued. In patients who have sustained VTE while taking TT, a laboratory evaluation for thrombophilia–hypofibrinolysis should be done. In patients with thrombophilia and a VTE event on TT, the TT should be stopped and not resumed, since recurrent VTE may occur despite adequate concurrent anticoagulation.

## Figures and Tables

**Table 1:** Serum testosterone (T), free T, and estradiol (E2) in 29 men before and on testosterone gel treatment (median 50 mg/day). TT: testosterone therapy.

	Normal Range	Mean ± SD	Low	Normal	High
**Before TT**					
T (ng/dL)	280–800	227 ± 124	66%	34%	0%
Free T (pg/mL)	7.2–24	7.5 ± 7.6	67%	30%	4%
E2 (pg/mL)	≤42.6	25.6 ± 11.9	87%		13%
**On TT**					
T (ng/dL)	280–800	431 ± 301	28%	62%	10
Free T (pg/mL)	7.2–24	17.7 ±17.2	36%	43%	21%
E2 (pg/mL)	≤42.6	30.3 ± 13.7	76%		24%

## Abbreviations

TT	testosterone therapy
VTE	venous thromboembolism
FVL	Factor V Leiden
PE	pulmonary embolism
